# Genetics of physiological dysregulation: findings from the long life family study using joint models

**DOI:** 10.18632/aging.102987

**Published:** 2020-04-01

**Authors:** Konstantin G. Arbeev, Olivia Bagley, Svetlana V. Ukraintseva, Deqing Wu, Hongzhe Duan, Alexander M. Kulminski, Eric Stallard, Kaare Christensen, Joseph H. Lee, Bharat Thyagarajan, Joseph M. Zmuda, Anatoliy I. Yashin

**Affiliations:** 1 Biodemography of Aging Research Unit, Social Science Research Institute, Duke University, Durham NC, 27708, USA; 2 Danish Aging Research Center, Department of Public Health, University of Southern Denmark 5000, Odense C, Denmark; 3 Taub Institute for Research on Alzheimer's Disease and the Aging Brain, Columbia University, New York, NY 10032, USA; 4 G. H. Sergievsky Center, Columbia University, New York, NY 10032, USA; 5 Departments of Epidemiology and Neurology, Columbia University Medical Center, New York, NY 10032, USA; 6 Department of Laboratory Medicine and Pathology, University of Minnesota, Minneapolis, MN 55455, USA; 7 Department of Epidemiology, University of Pittsburgh, Pittsburgh, PA 15261, USA

**Keywords:** aging, Mahalanobis distance, mortality, joint models, Long Life Family Study

## Abstract

Recently, Mahalanobis distance (D_M_) was suggested as a statistical measure of physiological dysregulation in aging individuals. We constructed D_M_ variants using sets of biomarkers collected at the two visits of the Long Life Family Study (LLFS) and performed joint analyses of longitudinal observations of D_M_ and follow-up mortality in LLFS using joint models. We found that D_M_ is significantly associated with mortality (hazard ratio per standard deviation: 1.31 [1.16, 1.48] to 2.22 [1.84, 2.67]) after controlling for age and other covariates. GWAS of random intercepts and slopes of D_M_ estimated from joint models found a genome-wide significant SNP (rs12652543, p=7.2×10^-9^) in the *TRIO* gene associated with the slope of D_M_ constructed from biomarkers declining in late life. Review of biological effects of genes corresponding to top SNPs from GWAS of D_M_ slopes revealed that these genes are broadly involved in cancer prognosis and axon guidance/synapse function. Although axon growth is mainly observed during early development, the axon guidance genes can function in adults and contribute to maintenance of neural circuits and synaptic plasticity. Our results indicate that decline in axons’ ability to maintain complex regulatory networks may potentially play an important role in the increase in physiological dysregulation during aging.

## INTRODUCTION

Aging is a complex process that involves multiple systems, leading to physiological dysregulation, health deterioration, and eventually death. Changes that occur at the molecular and cellular levels as individuals grow older propagate to changes detectable in laboratory tests of blood or other tissues and observable in measurements of various physiological variables in an individual at different ages. *Cross-sectional* measurements of such biomarkers correspond to the instantaneous profile of the current physiological state of an organism which provides valuable information about the current aging status of the body. Numerous studies show that such biomarkers are associated with risks of death and aging-related diseases (see, e.g., reviews in [1–3]). However, such “snapshots” of the physiological state do not help in understanding how exactly the organism arrived at this particular state. For example, if a person at some age has values of biomarkers that are associated with higher survival chances or reduced risks of diseases (e.g., lower values), it is unclear from the cross-sectional information alone if such outcome is due to lower values of respective biomarkers early in life, or due to their slower change with age, or both. Different studies have shown that dynamic characteristics of individual trajectories of biomarkers are associated with mortality risk and other aging-related traits [4]. To investigate such associations, one needs repeated measurements of biomarkers along with relevant time-to-event outcomes (e.g., mortality, onset of diseases) and other relevant health-related outcomes. Such information is routinely collected in contemporary longitudinal studies on humans and many of those, in addition, contain extensive genetic (and, most recently, various omics) data providing opportunities to explore this additional dimension in relation to aging, health, and longevity.

Analyses of longitudinal studies of aging present special methodological challenges due to inherent complexities that need to be taken into account to avoid biased inference. An essential assumption of such analyses is that the longitudinal outcomes (e.g., biomarkers) can be related to the risk of death so that the probability of having a missing value because of death depends on an unobserved value, which is missing not at random (MNAR) [5]. This means that standard methods such as mixed-effects models [6] or generalized estimating equations [7] are not appropriate in such applications because they assume the data are missing (completely) at random. Ignoring this can lead to severe bias, as is well-known in the statistical literature [8]. Furthermore, biomarkers are subject to measurement error and random biological variability; they can be collected at intermittent sparse examination visits, and typically they are not observed at event times. Ignoring measurement errors or biological variation and using the observed “raw” values of such variables as time-dependent covariates in the Cox regression model may lead to biased estimates and incorrect inferences [9, 10], especially when biomarkers are measured at sparse examinations or with a long time interval before an outcome event. Despite such evidence and well recognized needs for using appropriate methods in analyses of longitudinal data on aging [11–14], the adoption of such methods is slow.

**Figure 1:**
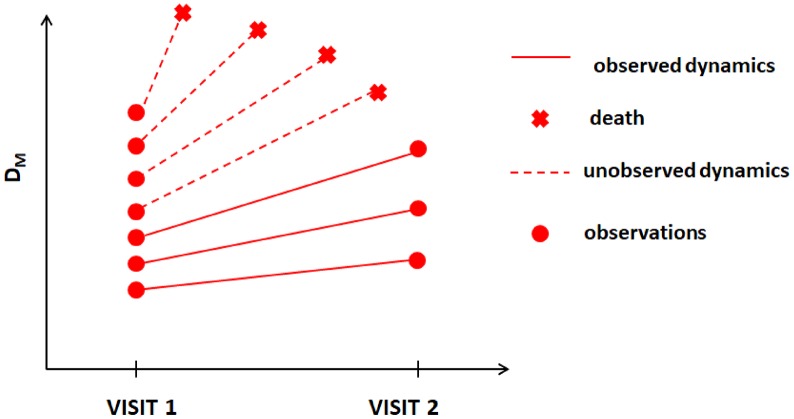
**Hypothetical dynamics of the measure of physiological dysregulation (D_M_) in LLFS among those who survived beyond visit 2 and those who died before visit 2.**

In this paper, we apply one such method developed for dealing with MNAR situations, joint models (JM) [15, 16], to data on mortality and available longitudinal measurements of multiple biomarkers from two visits in the Long Life Family Study (LLFS). We apply the statistical (Mahalanobis) distance measure (denoted as D_M_) [17] to reduce a high-dimensional biomarker space into a single measure that summarizes information about deviations of biomarkers from an optimal “baseline” state defined in a “reference population” and that is interpreted as the measure of physiological dysregulation [17]. D_M_ trajectories were shown to be good predictors of mortality, frailty, and chronic diseases in different studies [18–21] (with higher D_M_ values associated with higher mortality risk, etc.). The dynamic characteristics of D_M_ trajectories are related to different hidden mechanisms of aging-related changes that produce an increase in the risk of death with age [22], onset of unhealthy lifespan, and survival following the onset of unhealthy lifespan [23]. The LLFS collected follow-up data on mortality and measurements of multiple biomarkers in two visits as well as extensive data on common single nucleotide polymorphisms (SNPs) for genotyped participants. Such information allows one to construct D_M_ using biomarker data from both visits, to explore its dynamics in relation to mortality, and to perform genome-wide association studies (GWAS) to investigate genetic factors associated with such dynamics. However, the methodological complexities indicated above are applicable to this analysis. First, D_M_ is constructed from biomarkers that can have measurement errors and random biological variability and thus appropriate modelling (e.g., joint models [15, 16]) should be used rather than analyses using the observed “raw” values of D_M_ as time-dependent covariates in the Cox model [9, 10]. Second, the LLFS currently has only two visits (at which the biomarkers were collected) and many individuals died before visit 2; thus they will have D_M_ measured only at visit 1. D_M_ is known to be strongly associated with the risk of death [17, 18, 20, 22, 23]. Hence, individuals with adverse dynamics of D_M_ should tend to drop-out earlier due to death (see hypothetical illustration in Figure 1), i.e., the probability of drop-out can depend on missing (unobserved) values of D_M_. Also, a single observation at visit 1 does not provide information on the future dynamics of D_M_. However, information on time to death combined with available observations of D_M_ can still be used to infer the dynamics of D_M_ (if modeled appropriately). Here we illustrate this using such modelling (joint models) and show how the estimates from joint models can be used to perform GWAS to infer associations of SNPs with static and dynamic characteristics of the measure of physiological dysregulation (D_M_).

## RESULTS

### Empirical analyses and applications of joint models

Table 1 shows the characteristics of the LLFS sample (for the probands’ and offspring generations and the total sample) including information on variables used in fitting the joint models (see section Specification of joint models). Information on time-dependent variables is presented for each visit. Information on time-independent variables is given for each individual participating in the study (whether he/she was enrolled at baseline or at follow-up visit). See Notes under the table for the number of missing values for each variable.

**Table 1:** Characteristics of the LLFS Probands and Offspring generations and the total sample.

	**Probands**	**Offspring**	**Total Sample**
Number of participants at in-person visit 1	1673	3226	4899
Number of visit 1 participants who died before in-person visit 2	1050	189	1239
Number of visit 1 participants who returned for in-person visit 2	554	2623	3177
Number of new participants enrolled for in-person visit 2	14	123	137
Number of participants (returning and new) at in-person visit 2	568	2746	3314
Number of participants who died after in-person visit 2	173	43	216
Total number of participants in the study	1687	3349	5036
Number of deaths during the entire follow-up	1223	232	1455
Years from visit 1 to visit 2 (mean ± SD [range]) (for those with both visits)	7.13 ± 1.02 [5–11]	8.03 ± 1.1 [5–11]	7.88 ± 1.13 [5–11]
Age at visit 1 (mean ± SD [range])	89.57 ± 6.81 [49–110]	60.65 ± 8.37 [24–88]	70.53 ± 15.81 [24–110]
Age at visit 2 (mean ± SD [range])	92.93 ± 6.82 [56–110]	68.4 ± 7.84 [40–95]	72.39 ± 11.88 [40–110]
Females (%)	55.78	55.03	55.28
Participants from US field centers (%)	84.77	70.02	74.96
Low educated participants (below high school) (%)	24.72	6.42	12.55
Smokers (smoked >100 cigarettes in lifetime) (%)	37.7	45.6	42.95
Medication use at visit 1: anti-diabetic (%)	7.11	4.68	5.51
Medication use at visit 1: anti-hypertensive (%)	67.54	31.43	43.76
Medication use at visit 1: lipid-lowering (%)	31.68	25.33	27.5
Medication use at visit 2: anti-diabetic (%)	5.28	6.08	5.94
Medication use at visit 2: anti-hypertensive (%)	54.58	35.4	38.68
Medication use at visit 2: lipid-lowering (%)	33.27	29.53	30.18
Fasting (>=8 hrs.) at visit 1 (%)	88.11	89.77	89.2
Fasting (>=8 hrs.) at visit 2 (%)	54.05	74.25	70.79
Follow-up period (mean ± SD [range])	5.66 ± 2.97 [0–11.9]	8.36 ± 2.49 [0–12]	7.46 ± 2.95 [0–12]
Prevalence of cancer, number (%)	588 (34.85)	621 (18.54)	1209 (24.01)
Prevalence of CVD, number (%)	461 (27.33)	206 (6.15)	667 (13.24)
Prevalence of AD or dementia, number (%)	119 (7.05)	8 (0.24)	127 (2.52)
Prevalence of diabetes, number (%)	162 (9.60)	212 (6.33)	374 (7.43)
Incidence of cancer, number (%)	183 (10.85)	339 (10.12)	522 (10.37)
Incidence of CVD, number (%)	341 (20.21)	188 (5.61)	529 (10.50)
Incidence of AD or dementia, number (%)	119 (7.05)	27 (0.81)	146 (2.90)
Incidence of diabetes, number (%)	23 (1.36)	112 (3.34)	135 (2.68)

Note: Number of missing data (Probands, Offspring, Total Sample): indicator of death/lifespan - (0, 0, 0); age at visit 1 - (0, 0, 0); age at visit 2 - (100, 338, 438); sex - (0, 0, 0); country - (0, 0, 0); education - (8, 3, 11); smoking - (6, 14, 20); anti-diabetic drugs at visit 1 - (62, 424, 486); anti-diabetic drugs at visit 2 - (123, 529, 652); anti-hypertensive drugs at visit 1 - (62, 424, 486); anti-hypertensive drugs at visit 2 - (123, 529, 652); lipid-lowering drugs at visit 1 - (62, 424, 486); lipid-lowering drugs at visit 2 - (123, 529, 652); fasting at visit 1 - (3, 16, 19); fasting at visit 2 - (0, 0, 0); follow-up period - (0, 0, 0); cancer - (1, 3, 4); CVD - (1, 3, 4); AD - (5, 3, 8); diabetes - (4, 5, 9)

Table 1 also includes information on prevalent (existing before the baseline visit) and incident (new cases reported after the baseline visit) cases of major aging-related diseases in the LLFS. It shows that participants from the probands’ generation had higher prevalence of major diseases compared to the younger (offspring) generation, as expected. However, for incidence the pattern is not uniform: the proportions of new cancer cases are almost the same in two generations and the proportions of new diabetes cases tended to be higher in the offspring generation. We note also that verified information on causes of deaths of LLFS participants was not available for this study. Therefore, the numbers and proportions of individuals dying from different causes were not determined in this sample.

We constructed the measure of physiological dysregulation (denoted as $D_{M}^{1}$) from a set of biomarkers moderately correlated with age as described in Methods and separate measures based on subsets of respective biomarkers negatively and positively correlated with age ($D_{M}^{1-}$ and $D_{M}^{1+}$), as well as the “age-dependent” D_M_ variants (denoted, accordingly, $D_{Ma}^{1}$, $D_{Ma}^{1-}$, and $D_{Ma}^{1+}$) that considered deviations of biomarker values from those typical of age peers rather than those of younger individuals as in the original measures ($D_{M}^{1}$, $D_{M}^{1-}$, and $D_{M}^{1+}$) (see Methods). Table 2 lists the biomarkers used in these computations along with respective numbers of observations in each LLFS visit and the total number of observations.

**Table 2:** Biomarkers used in construction of statistical distance measures (D_M_).

**Name (Unit of Measurement)**	**Number of Observations Visit 1**	**Number of Observations Visit 2**	**Number of Observations Total**
Absolute monocyte count (10e9/L)	4,495	2,252	6,747
Creatinine (mg/dL)	4,658	2,615	7,273
**Diastolic blood pressure (mmHg)**	**4,757**	**2,777**	**7,534**
**Forced vital capacity (mL)**	**4,399**	**2,353**	**6,752**
**Grip strength (kg)**	**4,731**	**2,714**	**7,445**
**Hematocrit (%)**	**4,522**	**2,571**	**7,093**
Glycosylated hemoglobin (%)	4,626	2,596	7,222
Mean corpuscular volume (fl)	4,519	2,569	7,088
Pulse pressure (mmHg)	4,757	2,777	7,534
Red cell distribution width (%)	4,506	2,569	7,075
**Total cholesterol (mg/dL)**	**4,658**	**2,613**	**7,271**
White blood cell count (10e9/L)	4,521	2,555	7,076

Note: Biomarkers included in $D_{M}^{1-}$ (and its “age-dependent” variant $D_{Ma}^{1-}$) are highlighted in bold. Other (not highlighted) biomarkers are included in $D_{M}^{1+}$ and $D_{Ma}^{1+}$. All biomarkers indicated in the table are included in $D_{M}^{1}$ and $D_{Ma}^{1}$. See explanations about D_M_ variants in the text.

We compared the average values of D_M_ at visit 1 between (1) those who died between visit 1 and visit 2 and (2) those who survived beyond visit 2. The average values of D_M_ at visit 1 for those who died were significantly higher than those for the second group (see Table 3). Similar patterns were observed when stratified by sex, with larger and more significant differences for the “original” D_M_ variants. These analyses indicate that the situation depicted in Figure 1 is likely to be happening here, i.e., participants with higher values of D_M_ and/or higher rates of change tend to die earlier thus creating the paradigm for application of appropriate statistical techniques for joint analyses of longitudinal observations of D_M_ and time-to-event data on mortality.

**Table 3:** Average values of D_M_ at visit 1 among those who died between visit 1 and visit 2 and those who survived beyond visit 2 (standard deviations in parentheses).

**D_M_**	**Total Sample**			**Females**			**Males**		
	**Died before Visit 2**	**Alive at Visit 2**	**P-value**	**Died before Visit 2**	**Alive at Visit 2**	**P-value**	**Died before Visit 2**	**Alive at Visit 2**	**P-value**
$D_{M}^{1-}$	1.58 (0.42)	0.90 (0.48)	2.1×10^-254^	1.55 (0.42)	0.89 (0.47)	2.3×10^-116^	1.62 (0.42)	0.91 (0.50)	5.1×10^-138^
$D_{M}^{1+}$	1.34 (0.40)	0.97 (0.41)	1.7×10^-125^	1.38 (0.38)	0.98 (0.41)	8.3×10^-80^	1.31 (0.42)	0.97 (0.40)	3.1×10^-51^
$D_{M}^{1}$	1.51 (0.21)	1.15 (0.25)	3.4×10^-257^	1.51 (0.21)	1.15 (0.25)	1.6×10^-133^	1.50 (0.22)	1.16 (0.25)	4.1×10^-124^
$D_{Ma}^{1-}$	0.87 (0.49)	0.81 (0.47)	4.8×10^-4^	0.87 (0.50)	0.82 (0.47)	0.084	0.88 (0.47)	0.79 (0.47)	8.5×10^-4^
$D_{Ma}^{1+}$	0.92 (0.41)	0.85 (0.37)	5.7×10^-6^	0.93 (0.40)	0.85 (0.38)	4.6×10^-5^	0.90 (0.41)	0.86 (0.37)	0.018
$D_{Ma}^{1}$	1.14 (0.24)	1.09 (0.23)	3.2×10^-8^	1.15 (0.24)	1.09 (0.24)	1.4×10^-5^	1.14 (0.24)	1.09 (0.22)	4.5×10^-4^

Table 4 shows the results of application of the joint models to D_M_ variants ${\text{D}}_{\text{M}}^{1-}$, ${\text{D}}_{\text{M}}^{1+}$, and ${\text{D}}_{\text{M}}^{1}$ and mortality data in LLFS. The “Longitudinal” values represent estimates (coefficients, 95% confidence intervals [CI], and p-values for the null hypotheses of zero coefficients) from the fixed effects parts of joint models. Positive (and highly significant) values of the coefficient for age at visit 1 and follow-up period since visit 1 indicate that the trajectories of D_M_ increase with age for all variants, similar to other studies, e.g., [23]. The estimates for sex and country indicator varied in direction and significance showing that the levels of physiological dysregulation represented by the D_M_ variants constructed from different sets of biomarkers can be different in females and males and can have geographical/country-specific variation. Smoking and low education have consistent negative impacts on all D_M_ variants (i.e., smoking and low education are associated with an increase in the level of physiological dysregulation represented by the respective D_M_ variants). Use of medication for diabetes and hypertension, but not lipid-lowering medications also consistently showed negative impacts on the level of physiological dysregulation. The observations for anti-diabetic and anti-hypertensive drugs may reflect the destabilizing role of underlying disease in the physiological regulation since such drugs are typically prescribed after respective diagnoses. On the contrary, lipid-lowering drugs are commonly prescribed for prevention, often without clinical manifestation of a pathology (e.g., cardiovascular disease), which might contribute to their weaker association with physiological dysregulation. The fasting variable consistently reduced the values of D_M_, but it reached significance only for ${\text{D}}_{\text{M}}^{1}$. The “Survival” values represent estimates (coefficients, hazard ratios (HR) along with their 95% CI and p-values) from the survival parts of joint models. The results indicate that D_M_ (current value estimated continuously through time using the joint model) is associated with mortality (HR per standard deviation (SD) ranging from 1.66 for ${\text{D}}_{\text{M}}^{1+}$ to 2.22 for ${\text{D}}_{\text{M}}^{1}$) even after controlling for age and the other covariates indicated in the table (see Methods), all of which increase the risk of death with varying magnitude and significance levels.

**Table 4:** Results of the joint models applied to D_M_ variants $D_{M}^{1-}$, $D_{M}^{1+}$, and $D_{M}^{1}$.

**D_M_**	**Variable**		**Longitudinal**			**Survival**			
$D_{M}^{1-}$			**Coef.**	**95% CI**	**P-value**		**Coef.**	**HR**	**95 % CI**	**P-value**
	D_M_						1.380	2.10	[1.718, 2.577]	6.6×10^-13^
	AgeV1		0.023	[0.022, 0.024]	0		0.096	1.10	[1.089, 1.112]	6.7×10^-77^
	SexM		0.071	[0.047, 0.095]	7.9×10^-9^		0.239	1.27	[1.110, 1.453]	5.0×10^-4^
	IsDK		-0.117	[-0.147, -0.088]	8.9×10^-15^		0.486	1.63	[1.352, 1.954]	2.3×10^-7^
	LowEduc		0.063	[0.021, 0.105]	0.003		0.005	1.01	[0.851, 1.186]	0.955
	Smoke100		0.026	[0.002, 0.050]	0.036		0.134	1.14	[1.001, 1.305]	0.048
	DrugDiab		0.144	[0.097, 0.191]	2.4×10^-9^					
	DrugHtn		0.033	[0.009, 0.058]	0.008					
	DrugLipid		0.0003	[-0.025, 0.026]	0.983					
	Fasting		-0.026	[-0.065, 0.012]	0.183					
	TimeV1		0.017	[0.014, 0.020]	2.8×10^-35^					
$D_{M}^{1+}$										
	D_M_						1.190	1.66	[1.420, 1.937]	1.7×10^-10^
	AgeV1		0.011	[0.011, 0.012]	6×10^-177^		0.109	1.12	[1.107, 1.123]	4.6×10^-200^
	SexM		-0.013	[-0.034, 0.008]	0.221		0.385	1.47	[1.303, 1.658]	3.5×10^-10^
	IsDK		0.008	[-0.018, 0.034]	0.54		0.307	1.36	[1.159, 1.593]	1.6×10^-4^
	LowEduc		0.039	[0.003, 0.074]	0.034		0.024	1.02	[0.881, 1.192]	0.75
	Smoke100		0.026	[0.005, 0.047]	0.017		0.110	1.12	[0.989, 1.259]	0.075
	DrugDiab		0.303	[0.262, 0.344]	3.6×10^-47^					
	DrugHtn		0.068	[0.046, 0.089]	8.2×10^-10^					
	DrugLipid		-0.018	[-0.040, 0.004]	0.116					
	Fasting		-0.023	[-0.057, 0.011]	0.182					
	TimeV1		0.019	[0.016, 0.021]	4.5×10^-57^					
$D_{M}^{1}$										
	D_M_						2.883	2.22	[1.841, 2.673]	5.8×10^-17^
	AgeV1		0.011	[0.011, 0.012]	0		0.090	1.09	[1.084, 1.105]	2.4×10^-80^
	SexM		0.018	[0.006, 0.031]	0.005		0.317	1.37	[1.203, 1.568]	2.7×10^-6^
	IsDK		-0.027	[-0.042, -0.011]	6.2×10^-4^		0.373	1.45	[1.212, 1.739]	5.1×10^-5^
	LowEduc		0.042	[0.021, 0.064]	1.3×10^-4^		0.028	1.03	[0.870, 1.217]	0.741
	Smoke100		0.018	[0.005, 0.031]	0.005		0.077	1.08	[0.945, 1.235]	0.257
	DrugDiab		0.160	[0.136, 0.185]	3.4×10^-37^					
	DrugHtn		0.038	[0.025, 0.051]	7.1×10^-9^					
	DrugLipid		-0.001	[-0.014, 0.012]	0.922					
	Fasting		-0.025	[-0.045, -0.005]	0.016					
	TimeV1		0.014	[0.012, 0.015]	7.9×10^-81^					

Notes: 1.Variables: AgeV1: Age at visit 1, SexM: Sex (1 – male, 0 – female), IsDK: Participant is from Denmark (1) or USA (0), LowEduc: Low education (1 – below high school, 0 – otherwise), Smoke100: Smoker (1 – smoked >100 cigarettes in lifetime, 0 – otherwise), DrugDiab: Takes (1) or does not take (0) anti-diabetic drugs, DrugHtn: Takes (1) or does not take (0) anti-hypertensive drugs, DrugLipid: Takes (1) or does not take (0) lipid-lowering drugs, Fasting: Fasting (1 – >=8 hrs., 0 – otherwise), TimeV1: Follow-up period since visit 1.

2.Hazard ratios (HR) of all-cause mortality risk for D_M_ variables are per standard deviation: $D_{M}^{1-}$: 0.539, $D_{M}^{1+}$: 0.425, $D_{M}^{1}$: 0.276; HR for other variables are per a unit change.

For comparison, the results of application of the Cox model to the same data (with the respective D_M_ variant included as a time-dependent covariate carried forward from visit 1 and updated if there is a visit 2) are presented in Table 5. This table shows that the HRs for D_M_ from joint models were 1.3 to 1.4 times larger than the estimates from the Cox model.

**Table 5:** Results of the Cox model with D_M_ as a time-dependent covariate.

**D_M_**	**Variable**	**Coef.**	**HR**	**95 % CI**	**P-value**
$D_{M}^{1-}$					
	D_M_	0.877	1.60	[1.460, 1.764]	1.0×10^-22^
	AgeV1	0.105	1.11	[1.103, 1.120]	3.6×10^-162^
	SexM	0.275	1.32	[1.159, 1.494]	2.2×10^-5^
	IsDK	0.366	1.44	[1.219, 1.705]	1.9×10^-5^
	LowEduc	0.051	1.05	[0.901, 1.230]	0.517
	Smoke100	0.131	1.14	[1.005, 1.293]	0.041
$D_{M}^{1+}$					
	D_M_	0.478	1.23	[1.147, 1.309]	1.8×10^-9^
	AgeV1	0.121	1.13	[1.121, 1.136]	5.7×10^-296^
	SexM	0.386	1.47	[1.309, 1.655]	9.6×10^-11^
	IsDK	0.324	1.38	[1.184, 1.615]	4.2×10^-5^
	LowEduc	0.036	1.04	[0.897, 1.199]	0.626
	Smoke100	0.133	1.14	[1.016, 1.285]	0.026
$D_{M}^{1}$					
	D_M_	1.666	1.58	[1.442, 1.740]	7.4×10^-22^
	AgeV1	0.108	1.11	[1.106, 1.123]	2.7×10^-175^
	SexM	0.364	1.44	[1.269, 1.634]	1.7×10^-8^
	IsDK	0.360	1.43	[1.211, 1.698]	2.9×10^-5^
	LowEduc	0.055	1.06	[0.904, 1.236]	0.489
	Smoke100	0.105	1.11	[0.978, 1.261]	0.106

Notes: Variables: AgeV1: Age at visit 1, SexM: Sex (1 – male, 0 – female), IsDK: Participant is from Denmark (1) or USA (0), LowEduc: Low education (1 – below high school, 0 – otherwise), Smoke100: Smoker (1 – smoked >100 cigarettes in lifetime, 0 – otherwise).

Hazard ratios (HR) of all-cause mortality risk for D_M_ variables are per standard deviation: $D_{M}^{1-}$: 0.539, $D_{M}^{1+}$: 0.425, $D_{M}^{1}$: 0.276; HR for other variables are per a unit change.

The results for the “age-dependent” D_M_ variants ($D_{Ma}^{1-}$, $D_{Ma}^{1+}$, $D_{Ma}^{1}$) are shown in Tables 6 (joint models) and 7 (Cox model). In contrast to the “original” D_M_ variants ($D_{M}^{1-}$, $D_{M}^{1+}$, $D_{M}^{1}$), AgeV1, and TimeV1 have little influence on these measures (p=0.04 for $D_{Ma}^{1+}$, AgeV1; p=0.03 for $D_{Ma}^{1-}$, TimeV1; non-significant for the other cases; see Table 6, “Longitudinal”) reflecting the way such measures were constructed. Other variables in the “Longitudinal” part of Table 6 show varying significance and direction of impact on D_M_. The “Survival” part of Table 6 reveals that the “age-dependent” variants still have strong associations with mortality after controlling for age and the other covariates indicated in the table (see Methods) which showed significance levels similar to the original D_M_ variants. However, the effect size for “age-dependent” D_M_ variants diminished compared to the original D_M_ variants (HR per SD ranging from 1.31 for $D_{Ma}^{1-}$ to 1.7 for $D_{Ma}^{1}$). Table 7 shows that the HRs from joint models are also larger (1.1 to 1.4 times) than the HRs from the Cox model, similar to the original D_M_ variants.

**Table 6:** Results of the joint models applied to age-dependent D_M_ variants $D_{Ma}^{1-}$, $D_{Ma}^{1+}$, and $D_{Ma}^{1}$.

**D_M_**	**Variable**		**Longitudinal**				**Survival**			
$D_{Ma}^{1-}$			**Coef.**	**95% CI**	**P-value**		**Coef.**	**HR**	**95 % CI**	**P-value**
	D_M_						0.562	1.31	[1.155, 1.476]	2.1×10^-5^
	AgeV1		0.00006	[-0.001, 0.001]	0.904		0.125	1.13	[1.125, 1.140]	9.1×10^-300^
	SexM		0.003	[-0.024, 0.030]	0.821		0.364	1.44	[1.268, 1.634]	1.9×10^-8^
	IsDK		0.045	[0.012, 0.079]	0.008		0.255	1.29	[1.089, 1.530]	0.003
	LowEduc		0.030	[-0.018, 0.077]	0.221		0.066	1.07	[0.912, 1.251]	0.413
	Smoke100		0.037	[0.010, 0.065]	0.007		0.129	1.14	[1.000, 1.292]	0.049
	DrugDiab		0.100	[0.046, 0.154]	2.7×10^-8^					
	DrugHtn		0.011	[-0.017, 0.04]	0.427					
	DrugLipid		0.017	[-0.012, 0.046]	0.247					
	Fasting		-0.011	[-0.055, 0.033]	0.623					
	TimeV1		-0.003	[-0.006, -0.0003]	0.032					
$D_{Ma}^{1+}$										
	D_M_						1.332	1.67	[1.419, 1.965]	6.6×10^-10^
	AgeV1		-0.0008	[-0.001, -0.0004]	0.040		0.127	1.14	[1.129, 1.143]	0
	SexM		-0.0008	[-0.021, 0.020]	0.939		0.376	1.46	[1.290, 1.644]	1.3×10^-9^
	IsDK		0.013	[-0.013, 0.038]	0.325		0.282	1.33	[1.129, 1.556]	5.7×10^-4^
	LowEduc		0.041	[0.006, 0.077]	0.022		-0.001	.998	[0.856, 1.165]	0.986
	Smoke100		0.024	[0.003, 0.045]	0.024		0.112	1.12	[0.989, 1.263]	0.074
	DrugDiab		0.288	[0.247, 0.330]	8.1×10^-43^					
	DrugHtn		0.037	[0.016, 0.059]	7.3×10^-4^					
	DrugLipid		-0.033	[-0.055, -0.010]	0.004					
	Fasting		-0.029	[-0.063, 0.005]	0.098					
	TimeV1		-0.001	[-0.004, 0.001]	0.321					
$D_{Ma}^{1}$										
	D_M_						2.246	1.70	[1.445, 2.005]	1.9×10^-10^
	AgeV1		-0.0002	[-0.0007, 0.0003]	0.420		0.128	1.14	[1.129, 1.145]	4.2×10^-279^
	SexM		0.003	[-0.011, 0.016]	0.683		0.383	1.47	[1.286, 1.674]	1.2×10^-8^
	IsDK		0.010	[-0.006, 0.027]	0.223		0.279	1.32	[1.109, 1.576]	0.002
	LowEduc		0.030	[0.006, 0.054]	0.013		0.023	1.02	[0.866, 1.209]	0.791
	Smoke100		0.023	[0.01, 0.037]	7.3×10^-4^		0.088	1.09	[0.956, 1.247]	0.196
	DrugDiab		0.157	[0.130, 0.184]	1.0×10^-29^					
	DrugHtn		0.016	[0.002, 0.030]	0.024					
	DrugLipid		-0.012	[-0.026, 0.003]	0.114					
	Fasting		-0.019	[-0.042, 0.004]	0.103					
	TimeV1		-0.001	[-0.003, 0.001]	0.198					

Notes: Variables: AgeV1: Age at visit 1, SexM: Sex (1 – male, 0 – female), IsDK: Participant is from Denmark (1) or USA (0), LowEduc: Low education (1 – below high school, 0 – otherwise), Smoke100: Smoker (1 – smoked >100 cigarettes in lifetime, 0 – otherwise), DrugDiab: Takes (1) or does not take (0) anti-diabetic drugs, DrugHtn: Takes (1) or does not take (0) anti-hypertensive drugs, DrugLipid: Takes (1) or does not take (0) lipid-lowering drugs, Fasting: Fasting (1 – >=8 hrs., 0 – otherwise), TimeV1: Follow-up period since visit 1.

**Table 7:** Results of the Cox model with age-dependent D_M_ as a time-dependent covariate.

**D_M_**	**Variable**	**Coef.**	**HR**	**95 % CI**	**P-value**
$D_{Ma}^{1-}$					
	D_M_	0.284	1.14	[1.077, 1.216]	1.2×10^-5^
	AgeV1	0.126	1.13	[1.127, 1.142]	0.4×10^-309^
	SexM	0.375	1.46	[1.284, 1.650]	4.7×10^-9^
	IsDK	0.274	1.32	[1.112, 1.556]	0.001
	LowEduc	0.075	1.08	[0.922, 1.259]	0.348
	Smoke100	0.131	1.14	[1.004, 1.293]	0.043
$D_{Ma}^{1+}$					
	D_M_	0.503	1.21	[1.148, 1.283]	8.7×10^-12^
	AgeV1	0.128	1.14	[1.129, 1.143]	0
	SexM	0.376	1.46	[1.296, 1.637]	3.1×10^-10^
	IsDK	0.301	1.35	[1.158, 1.578]	1.4×10^-4^
	LowEduc	0.024	1.02	[0.885, 1.185]	0.748
	Smoke100	0.124	1.13	[1.006, 1.273]	0.04
$D_{Ma}^{1}$					
	D_M_	0.938	1.25	[1.173, 1.329]	3.1×10^-12^
	AgeV1	0.128	1.14	[1.129, 1.144]	0.6×10^-309^
	SexM	0.395	1.48	[1.307, 1.685]	1.1×10^-9^
	IsDK	0.284	1.33	[1.123, 1.572]	9.2×10^-4^
	LowEduc	0.067	1.07	[0.914, 1.252]	0.402
	Smoke100	0.100	1.11	[0.973, 1.255]	0.124

Notes: Variables: AgeV1: Age at visit 1, SexM: Sex (1 – male, 0 – female), IsDK: Participant is from Denmark (1) or USA (0), LowEduc: Low education (1 – below high school, 0 – otherwise), Smoke100: Smoker (1 – smoked >100 cigarettes in lifetime, 0 – otherwise). Hazard ratios (HR) of all-cause mortality risk for D_M_ variables are per standard deviation: $D_{Ma}^{1-}$: 0.474, $D_{Ma}^{1+}$: 0.385, $D_{Ma}^{1}$: 0.237; HR for other variables are per a unit change.

### Genetic analyses of D_M_-related traits

**Figure 2:**
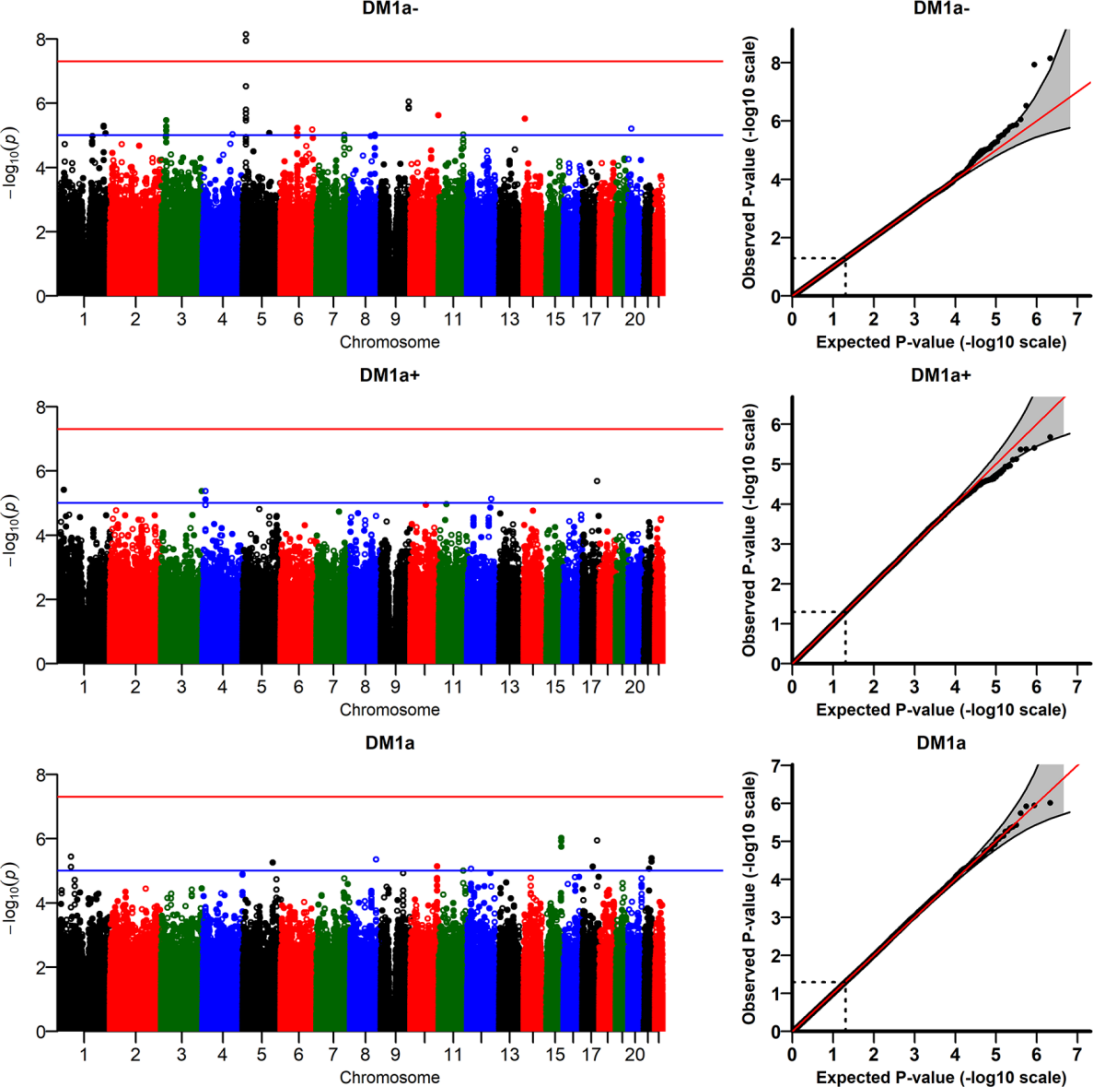
**Results of genome-wide association study of random slopes of D_M_ (D_M_-RS) for “age-dependent” D_M_ variants ($D_{Ma}^{1-}$, $D_{Ma}^{1+}$, $D_{Ma}^{1}$; see Methods).**

The R-package JM provides estimates of two types of random effects (random intercept, D_M_-RI, and random slope, D_M_-RS) for each individual in the analytic sample; these random effects are the traits used in the genetic analyses (see Methods). Figure 2 presents the results of the GWAS of $D_{M}$-RS for the "age-dependent" $D_{M}$ variants. We found two genome-wide significant variants on chromosome 5 in the *TRIO* gene (rs12652543, p=7.2×10^-9^, and rs16903264, p=1.2×10^-8^, which are in linkage disequilibrium (LD) [r^2^ ~ 0.95] in whites, according to the NIH-supported online tool *LDlink,* https://ldlink.nci.nih.gov) that are associated with $D_{M}$-RS for $D_{Ma}^{1-}$. Biological interpretations of these findings are provided in Discussion. Several SNPs from this region and some others showed suggestive associations (p < 10^-5^) with $D_{M}$-RI for $D_{Ma}^{1-}$ (Supplementary Figure 1). Analyses of other D_M_ variants (Figure 2: D_M_-RS for $D_{Ma}^{1+}$ and$D_{Ma}^{1}$; Supplementary Figure 2: D_M_-RS for $D_{M}^{1-}$, $D_{M}^{1+}$, and $D_{M}^{1}$; Supplementary Figure 3: D_M_-RI for $D_{M}^{1-}$, $D_{M}^{1+}$, and $D_{M}^{1}$) did not yield any genome-wide significant signals, but there were several suggestive signals on different chromosomes.

The D_M_ variants were constructed using several biomarkers (see Methods). To check whether the signals observed in the analyses of $D_{M}$-RS for $D_{Ma}^{1-}$ are due to associations of the genetic variants with some particular biomarker (or a subset of biomarkers) used in the construction of $D_{Ma}^{1-}$, we performed GWAS of $D_{M}$ constructed from individual biomarkers following the same procedures as for $D_{M}$ based on several biomarkers. The results are shown in Supplementary Figure 4. As one can see from this figure, there were no signals on chromosome 5 for any of the biomarkers constituting $D_{Ma}^{1-}$. However, the GWAS of $D_{M}$-RS for grip strength revealed a genome-wide significant signal (see description of sensitivity analyses below) on chromosome 7 (rs10231286, p=4.6×10^-8^; this SNP is located near *GRM3*, a glutamate receptor gene involved in brain functioning). Supplementary Table 1 shows top SNPs from this analysis.

We also performed sensitivity analyses running the models with different numbers of principal components (PCs: 1, 2, 5, 10). The top two signals for $D_{M}$-RS of $D_{Ma}^{1-}$ shown in Figure 2 remained genome-wide significant in all cases (p-values for the top SNP rs12652543 varied from p = 2.1 ×10^-8^ to p=9.7×10^-9^; p-values for the second-ranked SNP rs16903264 varied from p=3.3×10^-8^ to p=1.4×10^-8^). However, the genome-wide significant signal for grip strength found in the analyses with 20 PCs Hazard ratios (HR) of all-cause mortality risk for D_M_ variables are per standard deviation: $D_{Ma}^{1-}$: 0.474, $D_{Ma}^{1+}$: 0.385, $D_{Ma}^{1}$: 0.237; HR for other variables are per a unit change.

(Supplementary Figure 4) did not reach the genome-wide significant level in some models with different numbers of PCs (e.g., p=8.8×10^-8^ for the model with 2 PCs). In addition, we performed analyses of D_M_ variants constructed using different sets of biomarkers selected according to other thresholds for correlation with age (absolute value of the correlation ≥ 0.1 and ≥ 0.2) which did not produce any genome-wide significant signals. We also ran the model with time-interaction terms for all covariates (except age) in the fixed effects part of the longitudinal sub-model in JM. All interactions were non-significant except that for country (estimate: -0.008; p = 0.03). The GWAS results for $D_{M}$-RS of $D_{Ma}^{1-}$ constructed from this model were similar to those shown in Figure 2 (the top two SNPs remained genome-wide significant: p = 4.3×10^-9^ for rs12652543 and p = 7.4×10^-9^ for rs16903264).

## DISCUSSION

### Applications of joint models to composite measures of physiological dysregulation (D_M_) and genetic analyses of individual characteristics of D_M_, in the context of research on aging

In this work, we constructed the statistical (Mahalanobis) distance measure (D_M_) using multiple biomarkers collected at two visits in the LLFS using the original approach from [17] and its “age-dependent” modification that considers deviations of biomarker values from those typical of age peers rather than those of younger individuals. Analyses of longitudinal trajectories of such measures present methodological challenges (see Introduction) that require applying appropriate statistical methodology for correct inference. Here we applied one such method, joint models, for joint analyses of longitudinal observations of D_M_ and follow-up data on mortality for LLFS participants. Applications confirmed that, as in other studies [17, 18, 20, 22, 23], the association of D_M_ with mortality in LLFS is significant and effect sizes are substantial (with larger HRs for the “original” D_M_ variants). Comparisons of joint models with the Cox regression model with D_M_ considered as a time-dependent covariate indicated that the values of the association parameter for D_M_ in the hazard are underestimated in the Cox model, as expected [9, 10, 15]. Even though both models reveal the same direction of the influence and the result remains highly significant so that one may argue that the interpretation of the results in this case is the same in these two approaches, application of an inappropriate approach can still have substantial consequences if, for example, one needs to build a predictive model based on these results. As shown in many studies [4], including our applications to LLFS [24] and other data [23], inclusion of composite measures improves the predictive accuracy of the models for mortality and other health-related outcomes. Therefore, such predictive models should be based on an appropriate statistical approach such as joint models, which effectively account for informative missingness (death), and thus are able to correct for that type of bias.

Joint models is an active area of research in statistics with numerous extensions of the basic model (analyzed in this paper) suggested in the literature that cover a wide range of research applications such as latent classes, competing risks, multivariate models, non-linear models, dynamic predictions, stochastic processes, etc. (see books [15, 16] and recent review papers and tutorials [25–33]). Such extended models can be applied to analyze dynamic characteristics of composite measures such as D_M_ with various outcomes in more comprehensive ways.

One particular approach for joint analyses of longitudinal and time-to-event outcomes, the stochastic process model, SPM (see non-technical introduction in [4] and technical reviews in [34, 35]), is especially relevant in the context of research on aging as the model has components that permit clear biological interpretation in terms of fundamental features of aging-related changes in an organism. Our recent applications [22, 23] of SPM to D_M_ confirmed its significant association with mortality and proxy measures of physiological robustness and resilience, and revealed significant relationships of physiological dysregulation with other hidden aging-related characteristics, such as decline in stress resistance and adaptive capacity which typically are not observed in the data and thus can be analyzed only indirectly through such an analytic approach. The availability of genetic data in longitudinal studies makes it possible to explore genetic determinants of biological aging of individuals based on the dynamics of such composite measures as D_M_. However, this is still a largely unexplored area. The “genetic” SPM [36–38] allows investigation of genetic determinants of such aging-related characteristics in applications to longitudinal observations of composite biomarkers (such as D_M_). In particular, recent developments in the SPM methodology [39] have considerably enhanced the computational speed (which was a critical barrier in implementing this approach to large-scale genetic analyses) and opened new avenues for applying this model to GWAS, with far reaching implications for significantly improving our understanding of the genetic underpinnings of complex aging-related traits.

### Interpretation of genetic associations with D_M_: Biological and health effects of genes associated with the increase in physiological dysregulation with age

Table 8 shows the five genes (*TRIO*, *FNBP1*, *PLXNA4*, *CADM1*, and *UBE2E2*) corresponding to the top SNPs found in the association analysis of the random slope of D_M_ (for the “age-dependent” D_M_ constructed from biomarkers that decline in late life), including the SNP rs12652543 that reached genome-wide level of significance (other three genes shown in Table 8 will be discussed later). Note that multiple SNPs in these five genes were associated with slopes of D_M_, many of which were in LD with each other (according to *LDlink,* https://ldlink.nci.nih.gov). We eliminated the redundant SNPs (r^2^>0.8), so the SNPs shown in Table 8 represent not only themselves but also LD blocks (r^2^>0.8) of other (not shown) SNPs associated with D_M_ slopes. We then performed in depth review of scientific literature and information provided by the NCBI Gene (https://www.ncbi.nlm.nih.gov/gene/), and found that four of the above five genes have been implemented in cancer, especially in its progression/prognosis. Three of these genes (*TRIO*, *PLXNA4* [*Plexin A4*] and *CADM1* [*TSLC1, SynCAM1*]) are also involved in *axon guidance* and *growth* (Table 8, last column) [40–46].

**Table 8:** Top-ranked SNPs from GWAS of the random slope of D_M_, and respective genes (explanation in Discussion).

**SNP**	**Chr**	**Position**	**A1**	**A2**	**MAF**	**P-value**	**Region**	**Gene**	**Gene/protein is involved in**
rs12652543	5	14177235	A	G	0.18	7.2×10^-9^	intron	*TRIO*	cancer cells migration, invasion, prognosis, axon guidance,
rs32573	5	14172108	G	A	0.20	3.0×10^-7^	intron	*TRIO*	synapse function, neurite outgrowth, neurotransmission,
rs151473	5	14123313	T	C	0.16	1.6×10^-6^	20kb 5'	*TRIO*	cognition, intellectual disability
rs72757229	9	132653055	G	A	0.04	8.9×10^-7^	intron	*FNBP1*	high expression in cancer
rs79434268	7	131853832	A	G	0.07	9.8×10^-6^	intron	*PLXNA4 (Plexin A4)*	axon guidance, Parkinson's, AD, tau, cancer progression
rs1892773	11	115122626	C	A	0.21	9.6×10^-6^	intron	*CADM1 (TSLC1)*	synaptic cell adhesion, axon guidance, cancer prognosis
rs11713090	3	23570654	T	G	0.20	3.4×10^-6^	intron	*UBE2E2*	T2D
rs1436351	3	104617973	G	T	0.25	5.1×10^-5^	5' of	*ALCAM (CD166)*	cell adhesion, migration, cancer, axon growth, immunoglobulins
rs13097329	3	1320815	A	G	0.45	5.9×10^-5^	intron	*CNTN6*	cell adhesion, axon connections, intellectual disability
rs1444261	2	55354466	C	T	0.08	1.9×10^-5^	intron	*RTN4 (Reticulon4)*	nerve growth inhibitor, blocks regeneration

Notes: SNP – rs-number; Chr – chromosome number; Position – SNP position on chromosome; A1 – minor allele; A2 – major allele; MAF – minor allele frequency; P-value – p-value after GC; Region – SNP location in gene, or distance to closest gene; Gene – GENCODE gene name of closest gene; Gene/protein is involved in – cell process, biological function, or health disorder associated with this gene/gene product (based on the NCBI Gene resource [https://www.ncbi.nlm.nih.gov/gene/] and the up-to-date literature).

For a broader functional analysis, we selected 36 genes corresponding to the top 100 SNPs (all with p-value < 10^-4^) from GWAS of the D_M_-RS for the "age-dependent" D_M_ (Supplementary Table 2). We performed the pathway/process enrichment analyses for these 36 genes using several online tools available through Enrichr (https://amp.pharm.mssm.edu/Enrichr/ [47]) and MetaScape (http://metascape.org/gp/index.html#/main/step1) portals that exploit traditional ontologies and pathway sources, such as Gene Ontology [GO] processes, KEGG, BioCarta and Reactome pathway collections, among other. We also run the enrichment analysis using a commercial MetaCore platform for the functional analyses, by Clarivate Analytics (https://clarivate.com/products/metacore/), which uses custom-made manually curated libraries of pathways and processes, along with open-access ontologies, such as the GO and other [48]. We used several tools rather than just one since we wanted to feature pathways/processes that consistently show up among the top results of the enrichment analyses using the different tools.

We found that in most cases *axonal guidance* was among the top biological processes enriched for the 36 genes associated with the rate of increase in physiological dysregulation with age (D_M_-RS) (see examples in Supplementary Figure 5). These results were further supported by the information provided by NCBI Gene (https://www.ncbi.nlm.nih.gov/gene/) about biological effects of these 36 genes, and relevant research publications (e.g., [49–51]).

Then we used the Pathway Map Creator tool, a part of the MetaCore platform [48], to create a custom map showing only products of those genes (of the 36) that participate in functionally related biological processes (Supplementary Figure 6). This figure, again, pointed to a common involvement of *TRIO*, *PLXNA4* (Plexin A4) and *CADM1* (*TSLC1*) in axon guidance and growth, and in cell-cell adhesion, which plays a role in both the axon guidance and cancer, and also featured the products of three more genes among those 36 (*ALCAM* (*CD166*), *CNTN6* and *RTN4* (Reticulon 4)) as involved in the axon guidance and nerve growth. We added these three genes to Table 8, to show their biological effects in the context of the top significant genes.

In summary, our analysis of the biological effects of the top 36 genes from GWAS of the D_M_-RS, based on (i) the up-to-date scientific literature and the NCBI Gene resource, (ii) commercial (MetaCore) and open online pathway/process enrichment tools, and (iii) a custom pathway map creator (a part of the MetaCore platform), pointed to a common biological process that shows up across all these analyses, namely *axon guidance*. Although axon growth is mainly observed during early development, the axon guidance genes can be functional in adults and impact the maintenance of neural circuits, synaptic function and plasticity, neuroinflammatory responses, and as result neurological disorders [44, 52, 53]. Also, a recent study of the changes in human proteome across the lifespan revealed that proteins corresponding to genes involved in axon guidance and synaptic function are significantly over-represented among the clusters of proteins whose plasma levels show the strongest correlation with increasing age [54], thus supporting the role of respective biological processes in human aging.

Our results thus indicate that the decline in axons ability to form and maintain complex neuroregulatory networks may potentially play an important role in the increase in physiological dysregulation during aging.

In our recent paper we showed that the level of physiological dysregulation (estimated through D_M_) can be a useful aggregate indicator of the whole-body resilience and robustness [23]. In this context, our current results of the functional analysis of genetic associations with D_M_ may also indicate that the declining ability to form and maintain complex neuroregulatory networks could contribute to the decline in physical resilience with age, which is the key universal feature of aging [55]. This potential connection deserves further investigation.

One should note that our results do not imply that aging can be explained by a single biological process, such as the decline in axons ability to maintain complex networks that may lead to the increase in physiological dysregulation, in turn resulting in the decline in resilience and the increase in mortality risk with age. Aging is heterogeneous, and the increase in physiological dysregulation per se is one of potentially many processes contributing to its heterogeneity. Respectively, genes associated with the decline in physiological dysregulation are not the only genes involved in aging; however, they may significantly contribute to the genetic heterogeneity of aging.

### Concluding remarks

The “geroscience” hypothesis posits that interventions aimed at slowing biological aging could prevent or delay many different diseases simultaneously thus prolonging healthy lifespan and total lifespan [56]. Recent projections showed that the economic value of delayed aging (with a moderate increase in life expectancy by about 2.2 years, most of which would be spent in good health) is estimated to be $7.1 trillion over fifty years and, “in contrast, addressing major diseases such as heart disease and cancer separately would yield diminishing improvements in health and longevity by 2060 –- mainly due to competing risks” [57] providing additional arguments on the importance of identifying systemic factors that can underlie increased vulnerability to multiple diseases (rather than a specific pathology) in aging organisms. One of the critical barriers in developing interventions to slow or delay aging is that aging in humans is a gradual and slow process spanning years and decades which is not feasible to investigate in the timeframe of clinical trials. Thus, developing “proxy” measures quantifying the process of biological aging and investigation of the effects of different genetic and non-genetic factors on such measures is of paramount importance for moving research on aging forward.

Different measures to quantify biological aging (including D_M_) have been recently suggested in the literature and, as the recent comparative study of such measures reveals [58], they may not measure the same aspects of the aging process thus calling for further evaluation and refinement of such measures in additional studies. This, in particular, requires rich data containing relevant information on human aging and appropriate statistical methodology that would help utilize the full potential of such data. Our previous results [23] using Framingham Heart Study and Cardiovascular Health Study data suggested that multiple deviations of biomarkers from their baseline physiological states (reflected in higher physiological dysregulation levels summarized by D_M_) could be promising indicators of declining robustness and resilience during aging, and may precede clinical manifestation of not just one but many diseases (thus supporting a “geroscience” concept), even though deviations can be small and not clearly abnormal for individual biomarkers. The current paper is, to the best of our knowledge, the first study which revealed the significant genetic underpinnings of such composite measures of physiological dysregulation (D_M_) in the framework of the statistical approach relevant for joint analyses of longitudinal and time-to-event outcomes (joint models).

Results of GWAS of dynamic characteristics of D_M_ constructed from the output of joint models yielded genes (Table 8) broadly involved in the axon guidance, synaptic function, neuroinflammatory responses, cognitive disorders and cancer, which points out to a potentially important role of the decline in neurons ability to maintain complex regulatory networks in the increase in physiological dysregulation and related mortality risk during aging.

These encouraging findings call for further exploration of the genetic mechanisms of the change in physiological dysregulation with age, and its role in the heterogeneity of human aging. They also call for future replication in independent large cohorts that collect repeated measurements of biomarkers similar to those used in the construction of composite measures of physiological dysregulation in the LLFS data.

## MATERIALS AND METHODS

### Data

The Long Life Family Study (LLFS) is a family-based, longitudinal study of healthy aging and longevity that enrolled more than 4,900 participants from 583 families selected for exceptional familial longevity [59]. Participants were recruited at three U.S. (Boston, New York, Pittsburgh) and one European (Denmark) field centers during 2006–2009 based on age, capacity to understand the study, and their Family Longevity Selection Score (FLoSS) [59]. This score was developed specifically to select the families for the LLFS and it takes into account both the exceptionality of family members’ survival and the presence of very old living family members. The FLoSS was later validated in an independent large-scale genealogically-based resource (the Utah Population Database [60]) as a selection criterion for family longevity studies [61]. Sibships were eligible for the LLFS if their FLoSS was greater than 7 (this threshold was chosen because it was determined that such families are rare but are still detectable with sufficient frequency [59]) and they had at least one living sibling and at least one offspring willing to be enrolled in the study. Written informed consent was obtained from all subjects following protocols approved by the respective field center’s Institutional Review Boards. In this paper, we performed secondary analyses of LLFS data collected at all field centers. The data used in this study were provided by the LLFS Data Management and Coordinating Center (Washington University, St. Louis). The LLFS data are also available in the database of Genotypes and Phenotypes (https://www.ncbi.nlm.nih.gov/gap; Study Accession: phs000397.v1.p1).

Socio-demographic variables, data on past medical history and current medical conditions, medications use, physical and cognitive functioning, and blood samples were collected via in-person visits and phone questionnaires for all subjects at the time of enrollment, as described elsewhere [62]. Participants are followed-up annually to track their vital and health status. The analyses reported in this paper used the April, 20, 2018 release of LLFS data with the latest recorded date of death on January, 24, 2018. Ages at death for those participants who died within the follow-up period were computed from available dates of birth and death. Ages at censoring for those who did not die within the follow-up period were determined from dates of birth and last follow-up. The ages of the oldest participants were validated against external data [63]. Surviving participants underwent a second in-person evaluation in 2015–2018. Blood assays were centrally processed at a Laboratory Core (University of Minnesota) and protocols were standardized, monitored and coordinated through the LLFS Data Management and Coordinating Center. Genotyping was performed by the Center for Inherited Disease Research using Illumina Human Omni 2.5 v1 BeadChip array (see details on genotyping and quality control (QC) procedures in [64]).

We also reported disease-related characteristics of the analyzed sample that include the disease status at the baseline (prevalence) and new cases reported during the follow-up (incidence) for four major aging-related diseases available in the study: Alzheimer’s disease/dementia (AD), cancer, cardiovascular diseases (CVD), and diabetes. Information on diseases and health conditions was collected during the interviews either from the participants or proxies (if the participant was unable to respond). Using responses to questions about specific diseases (AD or dementia: Alzheimer’s Disease or Dementia; cancer: All cancer cites; CVD: Myocardial Infarction, Heart Attack, Coronary Angioplasty, Coronary Artery, Bypass Grafting, (Congestive) Heart Failure, Stroke, Cerebrovascular Accident, Transient Ischemic Attack, or Mini-Stroke; diabetes: Diabetes) from the baseline and the follow-up interviews, we computed the numbers of prevalent cases at the baseline and the numbers of new cases reported since the baseline.

### Construction of the measure of physiological dysregulation (D_M_)

The measure of physiological dysregulation (D_M_) is a recently developed approach for constructing a composite measure from multiple biomarkers [17, 18, 21]. It is a continuous measure which is essentially the statistical (Mahalanobis) distance [65] from “optimality” constructed for the joint distribution of multiple biomarkers and it uses the correlation structure of the biomarkers to measure how “aberrant each individual’s profile is with respect to the overall average (centroid) of the reference population” [19]. The “reference” centroid is assumed to represent the optimal physiological state. The “reference” population can be either a subsample of the same study population or it can come from some other study. For a set of biomarkers represented by a column vector *x* measured in an individual at age *t*, *x*(*t*), D_M_ is defined as [17]:

$$D_{M}(t)=\sqrt{{\left(x(t)-\bar{x}\right)}^{T}S^{-1}(x(t)-\bar{x})},$$ (1)

where $\bar{x}$ is a vector of means and *S* is the variance-covariance matrix for the respective biomarkers calculated from the “reference” population, and superscript *T* denotes transposition.

Information on biomarkers measured in the LLFS (number of measurements at each visit, correlations with age and pairwise correlations between biomarkers, p-values for testing the null hypothesis of a zero correlation, and number of observations used for computation of correlations) is given in Supplementary Table 3. For the purpose of this paper, we initially selected a set of biomarkers collected at both visits, for a total of 30 out of 47 biomarkers available in the study (see Supplementary Table 3). We then further reduced the list of biomarkers including only those moderately correlated with age (absolute value of the correlation ≥ 0.15; see description of sensitivity analyses for different correlation thresholds in Results) to consider for the computations of the statistical distance D_M_, following the ideas from previous work [17, 18]. Further, for the groups of related biomarkers (such as systolic/diastolic/pulse pressure; forced expiratory volume/forced vital capacity; red blood cell count/hematocrit/hemoglobin; total/low-density lipoprotein cholesterol; and white blood cell count/absolute neutrophil count), we randomly selected one biomarker for inclusion in D_M_. We constructed the D_M_ variants from the resulting set of biomarkers, separating those negatively and positively correlated with age, i.e., declining versus increasing at old ages (~65+) in most people [66]. Resulting variants were denoted $D_{M}^{1-}$ and $D_{M}^{1+}$, respectively. $D_{M}^{1-}$ includes the following biomarkers: diastolic blood pressure, forced vital capacity, grip strength, hematocrit, and total cholesterol, and $D_{M}^{1+}$ includes: absolute monocyte count, creatinine, glycosylated hemoglobin, mean corpuscular volume, pulse pressure, red cell distribution width, and white blood cell count. $D_{M}^{1}$ is computed using the combined list of biomarkers from $D_{M}^{1-}$ and $D_{M}^{1+}$.

We first constructed the D_M_ variants ($D_{M}^{1-}$, $D_{M}^{1+}$, $D_{M}^{1}$) using the conventional approach suggested in [17]. Specifically, the observed values of each biomarker were transformed using the Box-Cox transformation and standardized so that the transformed biomarkers were all on the same scale (with a zero mean and a unit variance). These standardized and transformed values were used in calculations of D_M_ as in Eq. 1. We used individuals aged <60 years at the LLFS visit 1 as a “reference population.” This cutoff gave a reasonable number of participants in the reference population: 1,407 (834 females, 573 males), 1,437 (847, 590), 1,389 (821, 568), for $D_{M}^{1-}$, $D_{M}^{1+}$, $D_{M}^{1}$, respectively. Computations of the means and variance-covariance matrix in the “reference” population were performed separately for females and males using the observed values of the biomarkers included in the definition of the respective D_M_. Note that in the LLFS there are at most two observations of biomarkers per individual. Therefore, we did not impute missing values of biomarkers as we did in our previous studies [22, 23] in applications to other datasets with longer series of measurements. Rather, the appropriate approach to handle missing not at random (MNAR) data is used here to jointly model the dynamics of D_M_ and the risk of death (see section Specification of joint models). For $D_{M}^{1-}$, we computed 6,244 values (4,056 at Visit 1, 2,188 at Visit 2) in 4,365 individuals. For $D_{M}^{1+}$, we calculated 6,557 (4,344, 2,213) values in 4,598 participants, and for $D_{M}^{1}$, the observations of 5,921 (4,010, 1,911) values in 4,290 persons were available. The resulting D_M_ variables were also transformed using the Box-Cox transformation. We performed sensitivity analyses (for the joint models analyses and genetic associations, see below) using the D_M_ in the original and the transformed scales which showed similar results so that only those for the Box-Cox transformed values are reported in the paper.

Next, we constructed the “age-dependent” D_M_ variants (denoted, accordingly, $D_{Ma}^{1-}$, $D_{Ma}^{1+}$, $D_{Ma}^{1}$) using a conceptually different approach in constructing the measure of physiological dysregulation. In the original specification [17], it was assumed that there is a fixed “normal” physiological state represented by the reference population (typically, a relatively younger sample) so that the D_M_ measures deviations from that state at respective ages. Here, in the definition of the “age-dependent” D_M_ we considered deviations of the biomarker values from those typical of age peers rather than those of younger individuals. That is, the means and variance-covariance matrix in Eq. 1 were defined in the respective age groups. Ideally, such values should be calculated for each age, however, due to small sizes of one-year age groups in our data, we computed these quantities for five-year age groups <50, 50-54, …, 90-94, 95+. For example, for a female aged 83 years at visit 1, the means and variance-covariance matrix were calculated based on a sample of females aged 80-84 years at visit 1 from which the D_M_ value at visit 1 was computed using the observations of biomarkers at visit 1 according to Eq. 1. Altogether, we computed 6,244 values (4,056 at Visit 1, 2,188 at Visit 2) for 4,365 individuals for $D_{Ma}^{1-}$, 6,557 (4,344, 2,213) values for 4,598 participants for $D_{Ma}^{1+}$, and 5,921 (4,010, 1,911) values for 4,290 persons for $D_{Ma}^{1}$.

### Specification of joint models

We used joint models [15, 16] as a tool to jointly estimate the longitudinal (D_M_) and time-to-event (mortality) outcomes (see Introduction). The R-package JM [67] version 1.4-8 was used to estimate the parameters of joint models. We applied the standard version of joint models as described below using the notations from [67]. The survival part of joint models quantifies the association between the longitudinal outcome and the risk of an event:

$$h_{i}(t|M_{i}(t),w_{i})=h_{0}(t)\exp\{\gamma^{T}w_{i}+\alpha m_{i}(t)\},$$ (2)

where $h_{i}(t|\cdot )$ is the hazard rate (mortality rate in our applications) for *i*-th individual at time point *t*, $M_{i}(t)=\{m_{i}(u),0\leq u<t\}$ denotes the history of the “true” (i.e., unobserved) longitudinal outcome ($m_{i}(\cdot )$, see below) up to *t*, $h_{0}(\cdot )$is the baseline hazard, $w_{i}$ is a vector of baseline covariates, γ is a corresponding vector of regression coefficients, and α is the parameter quantifying the effect of the longitudinal outcome on the risk of an event (this is usually the main parameter of interest in applications of joint models). A linear mixed effects model describes the dynamics of the longitudinal outcome (D_M_ in our case):

$$y_{i}(t)=m_{i}(t)+\varepsilon_{i}(t)=x_{i}^{T}(t)\beta +z_{i}^{T}(t)b_{i}+\varepsilon_{i}(t),$$ (3)

where $y_{i}(t)$ is the observed longitudinal process (with available observations $y_{ij}=\{y_{i}(t_{ij}),j=1\ldots n_{i}\}$ for *i*-th individual), β is a vector of fixed effects parameters, $b_{i}$ is a vector of random (normally distributed) effects, $x_{i}(t)$ and $z_{i}(t)$ are corresponding design vectors for fixed and random effects, and $\varepsilon_{i}(t)$ denotes a normally distributed error term (assumed to be independent of $b_{i}$) with zero mean and variance $\sigma^{2}$. The quantity $m_{i}(t)=x_{i}^{T}(t)\beta +z_{i}^{T}(t)b_{i}$ (that is, the observed value minus the error term) represents the “true” value of the longitudinal outcome which is included in the formula for the hazard rate (Eq. 2). This distinguishes joint models from the Cox model with time-dependent covariates which includes the observed values of the longitudinal process in the hazard rate. Ignoring measurement errors (or natural biological variation) in such variables in the latter model can result in underestimation of the strength of association between the hazard and the underlying longitudinal process [9, 10] which can be correctly inferred from joint models in such cases.

In our applications, the longitudinal trajectories of D_M_ (the longitudinal sub-model in joint models) were specified as a linear mixed effects model with linear (random intercept and random slope) random effects and time since visit 1 as a time variable (as implemented in the R-package JM). The list of covariates in the fixed effects part included: sex (1 – male, 0 – female), age at visit 1, country (1 – Denmark, 0 – USA), education (1 – below high school, 0 – otherwise), smoking (smoked >100 cigarettes in lifetime: yes [1]/no [0]), medication use (anti-diabetic, lipid-lowering, anti-hypertensive) (1 – used, 0 – did not use), and fasting (1 – ≥8 hours, 0 – otherwise). The groups of medications indicated above were constructed by the LLFS investigators in a separate study from original medications records using the corresponding Anatomical Therapeutic Chemical Classification System codes. We note that this list of medications does not include all possible groups of medications that might be relevant for this analysis (e.g., osteoporosis related medications). The time-to-event outcome (the survival sub-model in joint models) was modeled as the standard relative risk form [68] with the “true” or unobserved value of D_M_ (i.e., the estimate from the longitudinal sub-model [15]) included in the hazard (the “value” parameterization in the R-package JM, as in Eq. 2) along with the baseline covariates (the same list as above except medication use and fasting which are time-dependent covariates). The baseline hazard was represented as a piecewise constant function and the pseudo-adaptive Gauss-Hermite quadrature rule [69] was chosen to approximate the required integrals in the estimation algorithm (the "piecewise-PH-aGH" method in the R-package JM). We kept the default values for the number of internal knots (6 knots) in the baseline hazard and for the number of Gauss-Hermite quadrature points (3 points) used to approximate the integrals over the random effects. In some cases, the estimation algorithm in the R-package JM did not converge for the default values. In such situations, we varied the numbers (10 knots and/or 6 points) to achieve convergence. Sensitivity analyses confirmed that, in the cases of convergence, using models with different values for knots and points had little effect on the estimates of the parameter of interest (association parameter for D_M_ in the survival sub-model). See also sensitivity analyses with other specifications of JM described in Results.

### Genome-wide association study (GWAS) of D_M_-related traits

We performed GWAS of two D_M_-related traits constructed using the output of the joint models estimation procedure for the respective D_M_ variants specified above: random intercept (D_M_-RI) and random slope (D_M_-RS). These characteristics were computed for each study participant and define how the baseline value of D_M_ (D_M_-RI) and the age dynamics of D_M_ (D_M_-RS) in the particular individual differed from the average values in the study sample (adjusted for the respective covariates, as estimated by the fixed effects part of the longitudinal sub-model in joint models).

The QC procedure was performed according to the protocol described in [70] before running the association analyses. The original data contained 4,693 genotyped individuals (2,581 females, 2,112 males) and 2,225,478 SNPs. The sample QC check removed individuals with call rate below 95% and/or heterozygosity rate beyond ± 3 standard deviations (SD) from the mean as well as individuals of divergent ancestry (those for whom the first two principal components (PC) scores were beyond 8 SD from the respective mean scores for the HapMap Phase III European reference populations). The SNP’s QC check removed duplicated SNPs, variants with missing allele code information, indels, SNPs with call rate below 95%, minor allele frequency (MAF) below 1%, and those with a significant deviation from Hardy-Weinberg equilibrium (HWE) with p-value < 10^-5^. The resulting sample after QC contained 4,608 individuals of European ancestry (2,536 females, 2,072 males) and 1,464,300 autosomal SNPs which passed the QC procedure.

The R-package GENESIS (Bioconductor) [71] was used for the association testing and for computation of PCs using the PC-AiR method [72] to take into account the relatedness among individuals in the LLFS sample. The KING-robust kinship coefficient estimator [73] was used as the measure of ancestry divergence to identify a mutually unrelated and ancestry representative subset of individuals, as implemented in the PC-AiR algorithm. The mixed model was used for genetic association testing which included 20 PC-AiR PCs as fixed effects covariates to adjust for population stratification in the baseline scenario and a genetic relationship matrix (GRM) to account for genetic similarity among sample individuals. The kinship coefficient estimates from PC-Relate [74] (implemented in GENESIS) were used to construct the GRM. Genomic control (GC) [75, 76] was also applied as an additional tool to control for population stratification. In all analyses the GC lambdas were close to 1 indicating that the PCs were sufficient for this purpose.

We also performed sensitivity analyses to test the sensitivity of our results to various aspects of the analytic procedures. This included running GWAS for D_M_ variants computed from biomarkers selected using different thresholds for correlation with age, GWAS for separate biomarkers constituting the specific D_M_ variant, and analyses with different number of PCs, as described in Results.

## Supplementary Material

Supplementary Figures

Supplementary Tables 1 and 2

Supplementary Table 3

Supplementary References
