# Supplementary material for: Genetics of physiological dysregulation: findings from the long life family study using joint models
Source: Aging (Albany NY). 2020 Apr 1;12(7):5920–47. doi: 10.18632/aging.102987 (PMC7185144; doi:10.18632/aging.102987)
Supplement: Supplementary Figures [file aging-12-102987-s004..pdf]

SUPPLEMENTARY FIGURES

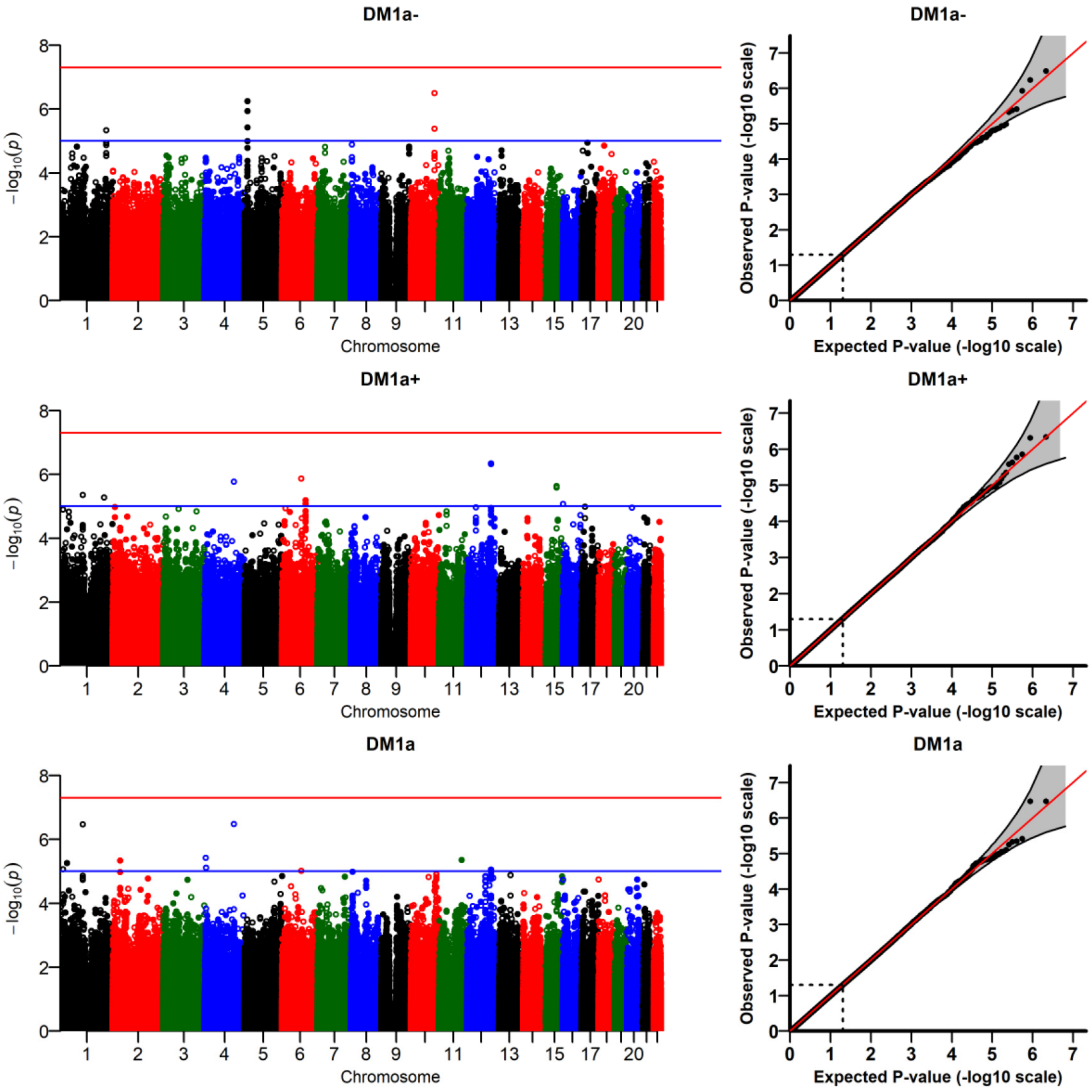

Supplementary Figure 1. Results of genome-wide association study of random intercepts of D_M (D_M-RI) for "age-dependent" D_M variants (D_{Ma}^{1-} , D_{Ma}^{1+} , D_{Ma}^{1} ; see Materials and Methods).

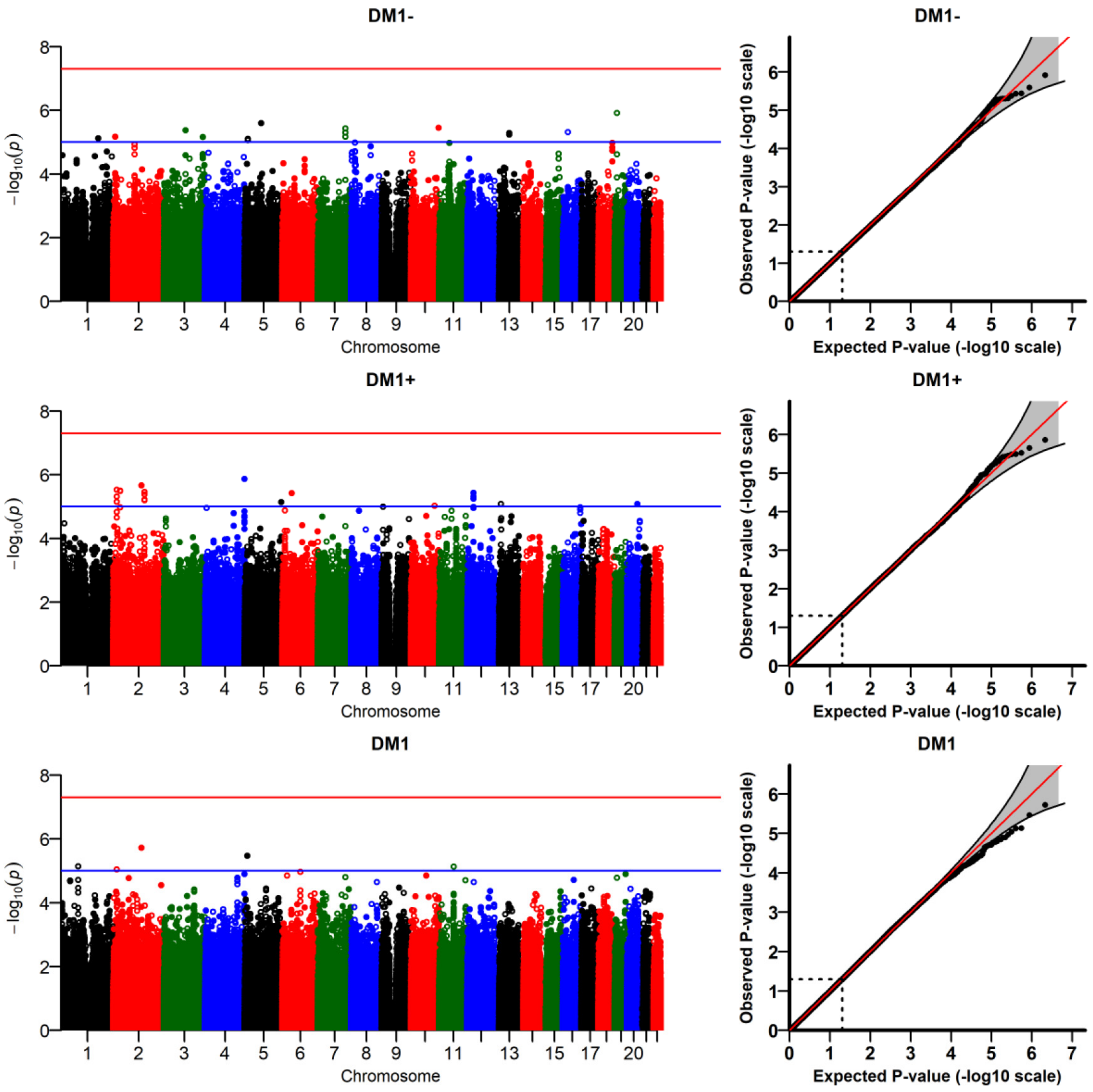

Supplementary Figure 2. Results of genome-wide association study of random slopes of D_M (D_M-RS) for “original” D_M variants (D_M^{1-} , D_M^{1+} , D_M^{1} ; see Materials and Methods).

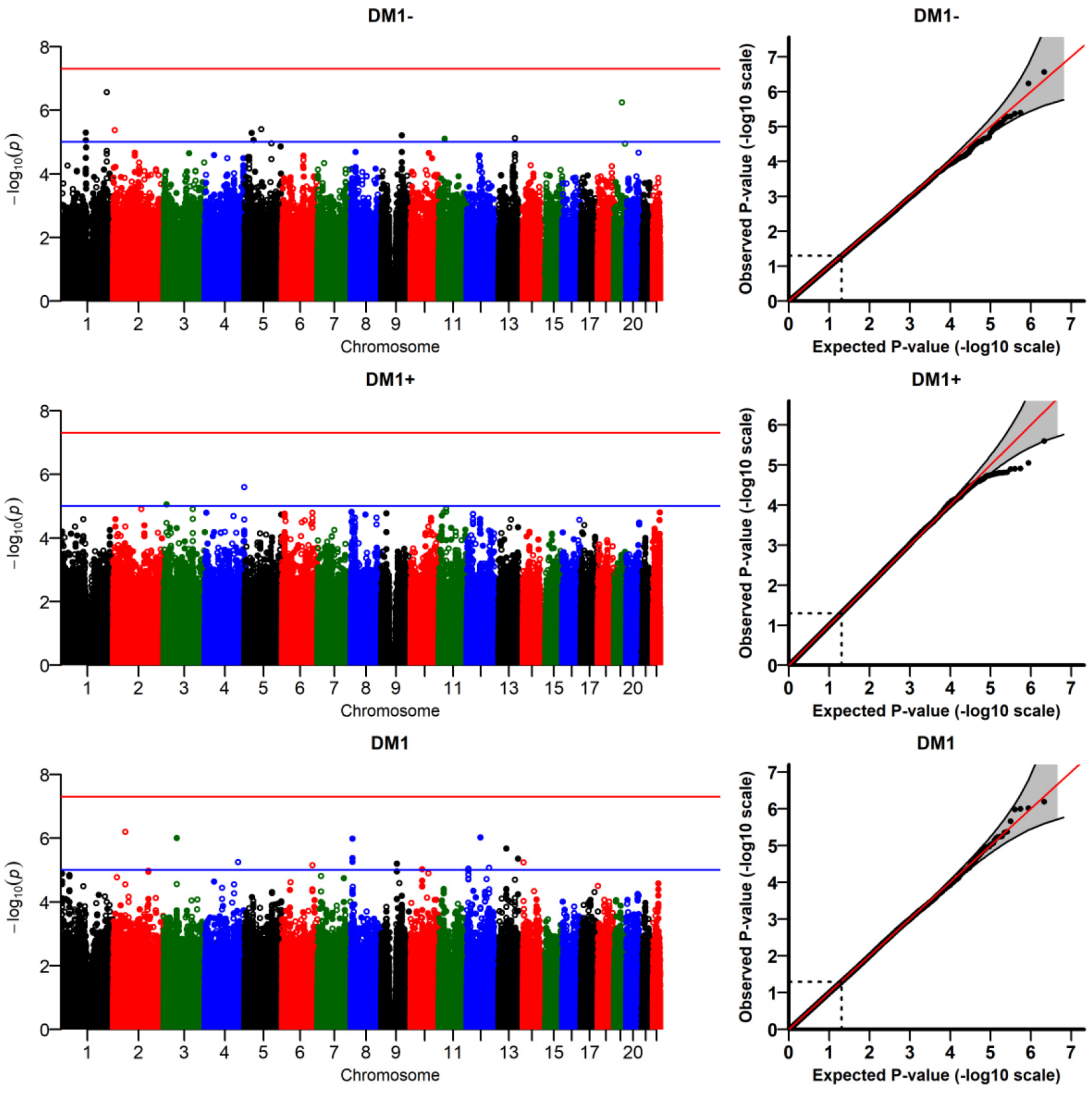

Supplementary Figure 3. Results of genome-wide association study of random intercepts of D_M (D_M-RI) for "original" D_M variants (D_M^{1-} , D_M^{1+} , D_M^{1} ; see Materials and Methods).

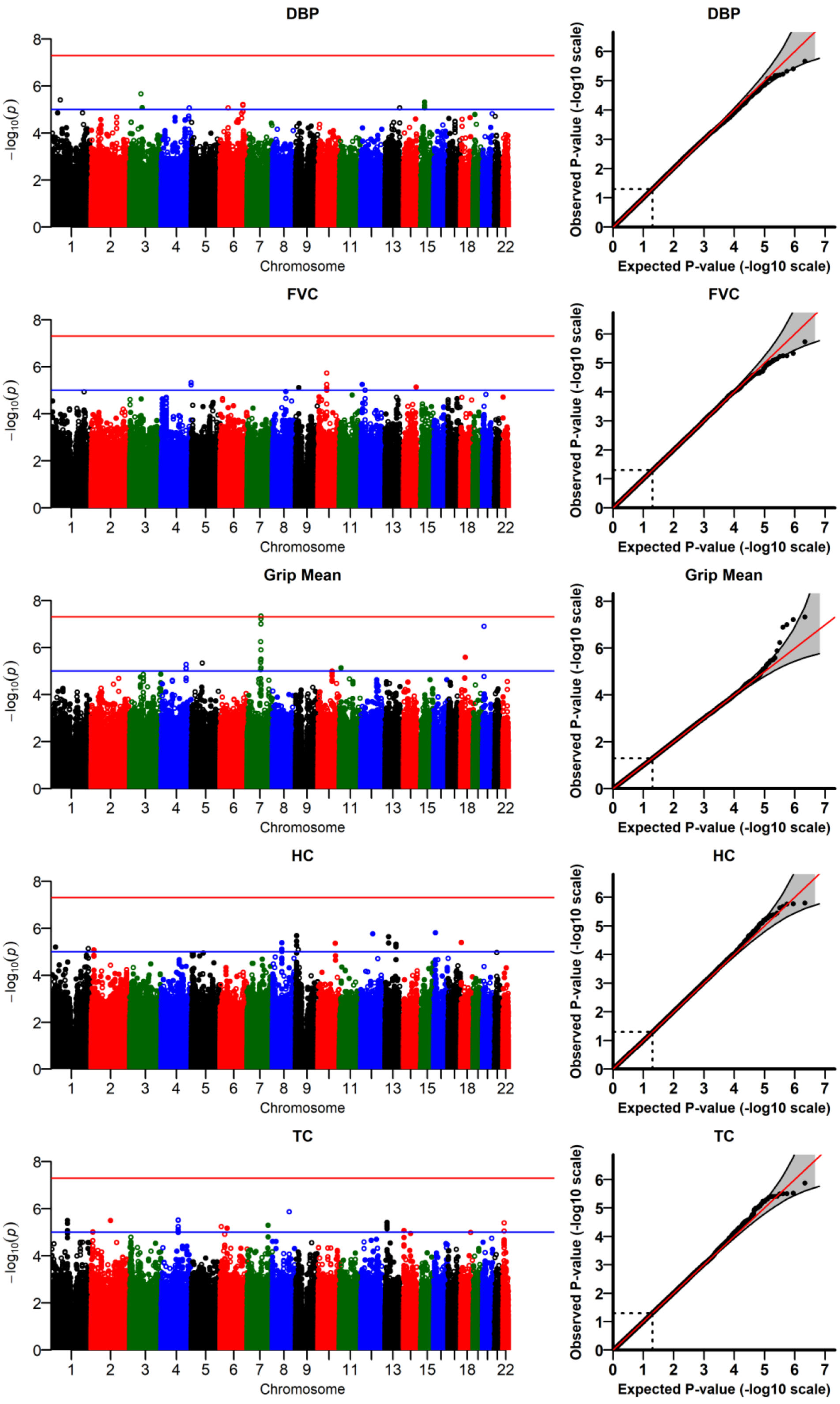

Supplementary Figure 4. Results of genome-wide association study of random slopes of D_M (D_M-RS) constructed for separate biomarkers constituting D_{Ma}^{1-} (see Materials and Methods). Notes: DBP: diastolic blood pressure; FVC: forced vital capacity; Grip Mean: mean grip strength; HC: hematocrit; TC: total cholesterol. For HC, the original scale of the biomarker was used due to non-convergence of joint model for the Box-Cox transformed scale.

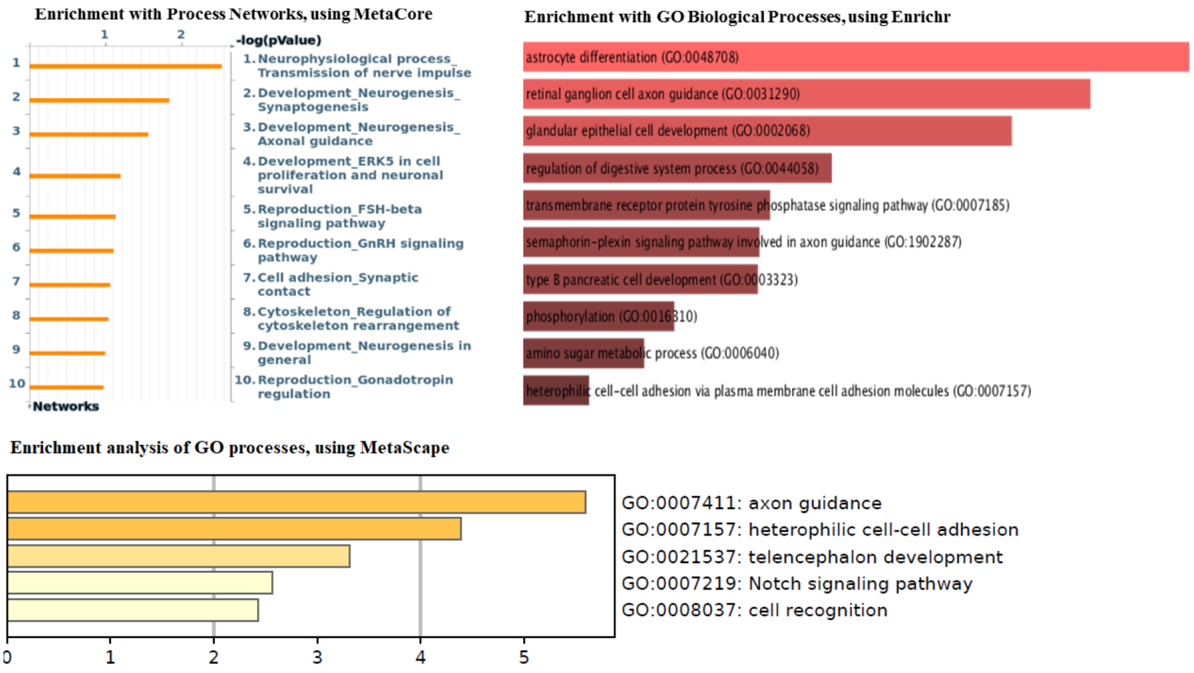

Supplementary Figure 5. Examples of pathway/process enrichment for the 36 genes corresponding to the top 100 SNPs from the GWAS of the D_M-RS for the "age-dependent" D_M, using different enrichment tools. Left top: Enrichment with Process Networks, using MetaCore [1]. N.B.: The content of these cellular/molecular processes is manually annotated and defined by Clarivate Analytics scientists (https://clarivate.com/products/metacore/). Right top: Enrichment with the Gene Ontology (GO) Biological Processes, using Enrichr (https://amp.pharm.mssm.edu/Enrichr/; [2]). Bottom: Enrichment with the Gene Ontology (GO) Processes, using MetaScape (http://metascape.org/gp/index.html#/main/step1).

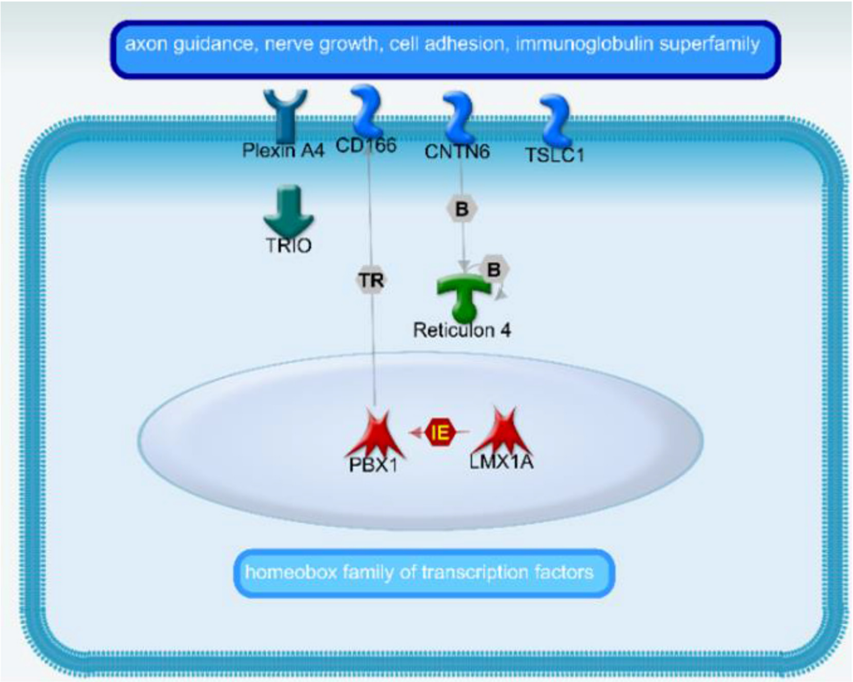

Supplementary Figure 6. Involvement of products of the genes associated with slopes of D_M in functionally related biological processes (explanation in Discussion).
