# Supplementary material for: Genetics of physiological dysregulation: findings from the long life family study using joint models
Source: Aging (Albany NY). 2020 Apr 1;12(7):5920–47. doi: 10.18632/aging.102987 (PMC7185144; doi:10.18632/aging.102987)
Supplement: Supplementary Tables 1 and 2 [file aging-12-102987-s001..pdf]

SUPPLEMENTARY TABLES

Supplementary Table 1. Top-ranked variants found in genome-wide association study of random slopes of D_M constructed for grip strength (see Supplementary Figure 4).

SNP	Chr	Position	A1	A2	MAF	P-val (GC)	Closest Gene	Gene Region
rs10231286	7	85967146	C	T	0.14	4.6×10^{-8}	GRM3	Intergenic/5' of GRM3
rs2037877	7	86066709	C	T	0.14	5.9×10^{-8}	GRM3	Intergenic/5' of GRM3
rs2373213	7	85990327	G	A	0.14	9.9×10^{-8}	GRM3	Intergenic/5' of GRM3
rs2423401	20	9649074	T	C	0.12	1.3×10^{-7}	PAK7	Intron
rs17160578	7	85672109	A	G	0.14	5.7×10^{-7}	GRM3	Intergenic/5' of GRM3
rs9969120	7	85990367	G	A	0.32	1.3×10^{-6}	GRM3	Intergenic/5' of GRM3
rs117882408	18	28482872	C	T	0.02	2.6×10^{-6}	DSC3	Intergenic/3' of DSC3
rs10255211	7	86124483	C	T	0.39	3.1×10^{-6}	GRM3	Intergenic/5' of GRM3
rs2708580	7	85890669	G	A	0.15	3.3×10^{-6}	GRM3	Intergenic/5' of GRM3
rs79576017	7	85744649	A	G	0.15	3.5×10^{-6}	GRM3	Intergenic/5' of GRM3
rs73382292	7	85730104	C	T	0.15	4.3×10^{-6}	GRM3	Intergenic/5' of GRM3
rs76915606	5	68769560	G	A	0.10	4.6×10^{-6}	OCLN	Intergenic/5' of OCLN
rs10030044	4	157011923	G	T	0.38	5.3×10^{-6}	CTSO	Intergenic/5' of CTSO
rs2708582	7	85893683	C	T	0.15	7.4×10^{-6}	GRM3	Intergenic/5' of GRM3
rs10769988	11	9071175	T	C	0.25	7.4×10^{-6}	SCUBE2	Intron
rs4417922	4	157006346	C	T	0.32	7.8×10^{-6}	CTSO	Intergenic/5' of CTSO
rs11722256	4	156994724	T	G	0.32	8.0×10^{-6}	CTSO	Intergenic/5' of CTSO
rs12668989	7	85662201	C	T	0.15	8.9×10^{-6}	GRM3	Intergenic/5' of GRM3
rs11201991	10	88117122	C	T	0.30	1.0×10^{-5}	GRID1	Intron
rs7904985	10	88116479	A	G	0.30	1.2×10^{-5}	GRID1	Intron

Supplementary Table 2. The list of the thirty-six genes (in alphabetical order) corresponding to the top 100 SNPs found in GWAS of the D_M-RS.

7SK, AGAP1, ALCAM, ANO4, BMP2K, C2orf48, CADM1, CDC42BPA, CDH4, CDK15, CDK6, CNTN6, EIF3H, ELMOD1, FNBP1, GPC5, GRIK2, LEUTX, LMX1A, MACROD2, NBEAL2, PBX1, PCDH9, PLXNA4, RNF150, RSPO4, RTN4, SGK1, ST6GAL1, STXBP6, SYNE1, TCERG1L, THSD7B, TRIO, UBE2E2, UTP23.

Supplementary Table 3. Information on biomarkers measured in the LLFS (number of measurements at each visit, correlations with age and pairwise correlations between biomarkers, p-values for testing the null hypothesis of a zero correlation, and number of observations used for computation of correlations).
