# Supplementary material for: Genetics of physiological dysregulation: findings from the long life family study using joint models
Source: Aging (Albany NY). 2020 Apr 1;12(7):5920–47. doi: 10.18632/aging.102987 (PMC7185144; doi:10.18632/aging.102987)
Supplement: Supplementary References [file aging-12-102987-s003..pdf]

SUPPLEMENTARY REFERENCES

1. Dubovenko A, Nikolsky Y, Rakhmatulin E and Nikolskaya T. Functional Analysis of OMICs Data and Small Molecule Compounds in an Integrated "Knowledge-Based" Platform. In: Tatarinova TV and Nikolsky Y, eds. Biological Networks and Pathway Analysis. (New York, NY: Springer New York), 2017. pp. 101–124. https://doi.org/10.1007/978-1-60761-175-2_10

2. Kuleshov MV, Jones MR, Rouillard AD, Fernandez NF, Duan Q, Wang Z, Koplev S, Jenkins SL, Jagodnik KM, Lachmann A, McDermott MG, Monteiro CD, Gundersen GW, Ma'ayan A. Enrichr: a comprehensive gene set enrichment analysis web server 2016 update. Nucleic Acids Res. 2016; 44:W90–7. https://doi.org/10.1093/nar/gkw377 PMID:27141961
